# Changing Trends in the Fungal Aetiology of Chronic Suppurative Otitis Media and Antifungal Susceptibility Patterns of Isolated Fungi: A Pilot Study From a Tertiary Care Centre in Central India

**DOI:** 10.7759/cureus.105680

**Published:** 2026-03-23

**Authors:** Arati A Bhadade, Farha Siddiqui, Utkal Mishra, Shaila Sidam, Rashma Binu, Madhu Panthi, Ankita beniwal, Tinku K Chaubey, Vikas Lal, Karuna Tadepalli

**Affiliations:** 1 Department of Microbiology, All India Institute of Medical Sciences, Bhopal, Bhopal, IND; 2 Department of Otorhinolaryngology - Head and Neck Surgery, All India Institute of Medical Sciences, Bhopal, Bhopal, IND; 3 Department of General Surgery, All India Institute of Medical Sciences, Bhopal, Bhopal, IND

**Keywords:** antifungal drug resistance, antifungal susceptibility testing (afst), candida auris, chronic suppurative otitis media (csom), fungal infection, trichomonascus ciferrii

## Abstract

Introduction

Middle-ear and mastoid mucosal inflammation that persists for at least six weeks with discharge is known as chronic suppurative otitis media (CSOM). It is prevalent in India, and fungal infections contribute to a notable proportion of cases. Irrational use of steroids and antibiotics is associated with increased fungal isolates. This study aims to evaluate the presence, profile, and antifungal susceptibility (AFST) of fungi in CSOM patients, using Sensititre™ YeastOne® (Thermo Fisher Scientific, Waltham, MA, USA) colorimetric broth microdilution for yeasts and disk diffusion for moulds, although bacterial causes are well established.

Materials and methods

This study was a one-year cross-sectional study conducted in the Department of Microbiology in collaboration with the Departments of Microbiology and ENT-Head & Neck Surgery. This one-year cross-sectional study included patients aged 12 years or older with purulent ear discharge lasting at least six weeks. Samples of middle ear exudate were gathered and prepared for mounting and culture in 10%-20% potassium hydroxide (KOH). Isolates of yeast or mould were identified at the species level using Matrix-Assisted Laser Desorption/Ionization Time-of-Flight Mass Spectrometry (MALDI-TOF MS; Bruker Daltonics, Bremen, Germany). For the isolated yeasts, AFST was performed using the Sensititre™ YeastOne® colourimetric broth microdilution method in accordance with the CLSI M27-A3 standard, and results were interpreted according to the M60 guideline. The data were collected in an Excel sheet (Microsoft® Corp., Redmond, WA, USA), and the statistical analysis involved both descriptive and inferential techniques.

Results

A total of 136 clinical samples were analyzed in the study. Of these, 27 (19.9%) were KOH positive, including 12 (44.4%) that were culture negative. Overall, 30 samples (22.1%) were culture positive, of which 15 (50%) were KOH negative. The overall fungal prevalence in the study was 42 (30.9%). Among the 30 culture-positive cases, six (20%) were moulds, and 24 (80%) were yeasts. *Candida albicans* (n = 9) was less common than non-albicans *Candida* (NAC) isolates (n = 15); however, it was the most prevalent single yeast species among culture isolates (30%). *Trichomonascus ciferrii* and *Candida auris* each had four (13.3%) isolates. *Candida orthopsilosis* (3, 10.0%), *Candida metapsilosis* (2, 6.7%), and *Candida tropicalis* (2, 6.7%) were among the other yeasts found. *Aspergillus fumigatus* was the most prevalent species among mould isolates (3, 10.0%), followed by *Aspergillus flavus* (1, 3.3%), *Aspergillus niger* (1, 3.3%), and *Fusarium solani* (1, 3.3%). After performing AFST on yeast isolates, most isolates showed resistance to one or more antifungal drugs tested, particularly *C. auris* and *T. ciferrii* isolates.

Conclusion

There is currently a change in the fungal etiological pattern in CSOM patients, with increased isolation of new species, including *T. ciferrii*, *C. auris*, *C. orthopsilosis*, and *C. metapsilosis*. Identification of fungi to the species level is crucial for treatment, as many yeast species have demonstrated resistance to one or more antifungal drugs. Echinocandins and amphotericin B continue to work, but yeasts - particularly NAC species, like *C. auris* and *T. ciferrii - *often exhibit significant azole resistance.

## Introduction

Ear discharge that lasts for at least six weeks through a perforated tympanic membrane, which may occur from spontaneous rupture, is a hallmark of chronic suppurative otitis media (CSOM), a chronic infection of the middle ear and mastoid mucosa [1]. Serious consequences, such as irreversible hearing loss and, in rare instances, potentially fatal intracranial or extracranial sequelae, can result from CSOM if treatment is not received [2,3]. In low- and middle-income nations, the issue is particularly pronounced, and the burden remains heavy in India. According to localized community studies, the prevalence of CSOM in India varies but is disturbingly high, despite the World Health Organization's (WHO) estimate of 7.8% [4].

Recent studies highlight the shifting microbiology of this condition, with fungal aetiologies increasingly identified alongside traditional bacterial pathogens. For example, a study in a low- to middle-income setting reported fungi in about 28.6% of CSOM cases, with *Aspergillus* spp. (≈50%) and *Candida* spp. (≈29%) being the most common isolates. This is consistent with previous research showing that the estimated fungal infection rates in CSOM average about 24% and range from 3.9% to 49% [5,6]. Alkaline epithelial debris buildup and the chronic wet environment of continuous ear discharge promote fungal proliferation in CSOM by providing favourable conditions for fungal colonization.

Several aggravating factors increase the risk of fungal superinfection. When topical antibiotics are overused, often in combination with steroids, the natural microbial ecology of the ear may be disrupted, and opportunistic fungi may inadvertently flourish. Few recent studies have thoroughly examined fungal prevalence or resistance profiles in CSOM patients in India, despite the increased recognition of fungal involvement. The majority of CSOM research still focuses on aerobic bacteria.

This changing circumstance highlights the necessity for more focused research. It is particularly important to evaluate the current incidence of fungal CSOM and to understand trends in antifungal susceptibility (AFST) among fungal isolates. Assessing resistance patterns is crucial, given reports of increasing azole resistance among *Candida* spp., particularly non-albicans *Candida* (NAC), in India, although amphotericin B generally remains effective against most isolates [7,8].

This pilot study aims to fill existing information gaps about these dynamics by (i) determining the incidence of fungal pathogens among CSOM patients and (ii) characterizing the AFST profiles of the isolated fungal species. By shedding light on the changing fungal aetiology of CSOM and potential trends in antifungal resistance, our findings can guide more cautious antifungal medication use and enhance clinical outcomes for patients with this chronic ear condition.

## Materials and methods

Study design and setting

In collaboration with the ENT Department at a 950-bed tertiary care academic hospital in Central India, the Microbiology Department conducted a 12-month hospital-based prospective cross-sectional study from July 2024 to July 2025. With Institutional Ethics Committee approval, this study was conducted, and all ethical guidelines were followed (approval no. IHEC-LOP/2024/P24/039).

Study population and sample size

The study was a prospective cross-sectional study conducted over a period of one year and included patients aged 12 years or older who presented to the ENT Outpatient Department during the study period with clinical symptoms of CSOM, including persistent purulent ear discharge lasting six weeks or longer. Patients with a history of surgical treatment for ear infections, non-consenting patients, and those with recalcitrant disease, defined as CSOM persisting despite adequate medical therapy for at least six weeks or requiring repeated courses of treatment, were excluded. Written informed consent was obtained before recruitment for the study. As this was a pilot study, no formal sample size calculation was performed; the inclusion of 136 clinical samples was based on feasibility and the number of eligible patients presenting during the study period.

Sample collection procedure

**Figure 1:**
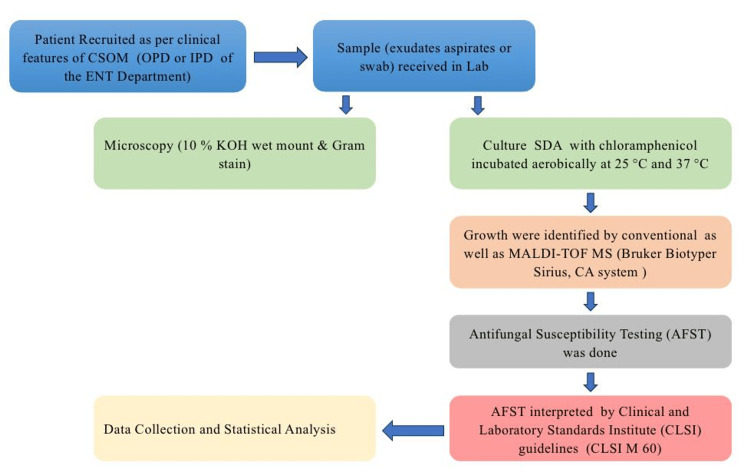
Work flow of the study. This image was created by Dr. Farha Siddiqui using Microsoft PowerPoint (Microsoft Corporation, Redmond, WA, USA). CSOM: Chronic Suppurative Otitis Media; MALDI-TOF MS: Matrix-Assisted Laser Desorption/Ionization Time-of-Flight Mass Spectrometry; SDA: Sabouraud's Dextrose Agar

Following acceptance, sterile cotton swabs, 70% alcohol solution, and povidone-iodine were used to clean each patient's external auditory canal (EAC). To reduce contamination, the canal was then allowed to dry for three minutes. A sterile syringe needle was used to aspirate middle ear exudates through the perforated tympanic membrane while the area was illuminated. If aspiration was not feasible, swabs were taken instead. During the aspiration procedure, precautions were taken to prevent contamination from the EAC. For a fungal culture test, the gathered samples were promptly taken to the microbiology lab (Figure 1), demonstrating the sample collection and processing method.

Sample processing

Microscopy

Gram staining and a 10% potassium hydroxide (KOH) wet mount test were performed on all samples included in the study to detect fungal elements.

Culture

Regardless of the microscopy findings, all samples were inoculated on two Sabouraud's Dextrose Agar (SDA) plates with chloramphenicol and incubated aerobically at 25°C and 37°C until growth was detected or for four weeks to report them as sterile. Fungal isolates (yeast and mould) were identified using both conventional methods as well as by Matrix-Assisted Laser Desorption/Ionization Time-of-Flight Mass Spectrometry (MALDI-TOF MS; Bruker Daltonics, Bremen, Germany). Cases with fungal elements detected by microscopy, or culture alone, or both, were considered to have a fungal aetiology contributing to CSOM. For patients in whom moulds were cultured, repeat samples were tested to confirm pathogenicity.

Antifungal Susceptibility Testing (AFST)

For yeast isolates, AFST was performed using the Sensititre™ YeastOne® (Thermo Fisher Scientific, Waltham, MA, USA) colorimetric method for broth microdilution [9], according to the manufacturer's guidelines and the Clinical and Laboratory Standards Institute (CLSI) guidelines M27 and M38 [10,11]. The results were interpreted based on the latest CLSI-M60 [12] and available epidemiological cutoff values (ECVs), for which breakpoints are not available; however, the ECVs have been established in wild-type (WT) and non-wild-type (NWT) strains, and they were interpreted for posaconazole, itraconazole, flucytosine, and amphotericin B (except for *Candida auris*) [13]. The geometric mean (GM) was computed for the antifungal for which neither the breakpoint nor the ECV is provided. Nine different antifungal medications were examined, including 5-flucytosine; azoles (anidulafungin, caspofungin, and micafungin); polyene (amphotericin B); and azoles (fluconazole, posaconazole, itraconazole, and voriconazole). The disc diffusion method was used to evaluate the AFST of mould isolates using voriconazole (5 µg).

Data collection and statistical analysis

Data entry was performed using Microsoft Excel (Microsoft® Corp., Redmond, WA, USA) to systematically record all relevant information for each participant, facilitating analysis. Categorical variables were expressed as frequencies and percentages, while continuous variables were presented as mean ± standard deviation. The Chi-square test was used to assess associations between categorical variables, whereas Student’s t-test was applied to compare continuous variables between groups as appropriate. A p-value < 0.05 was considered statistically significant.

## Results

**Figure 2:**
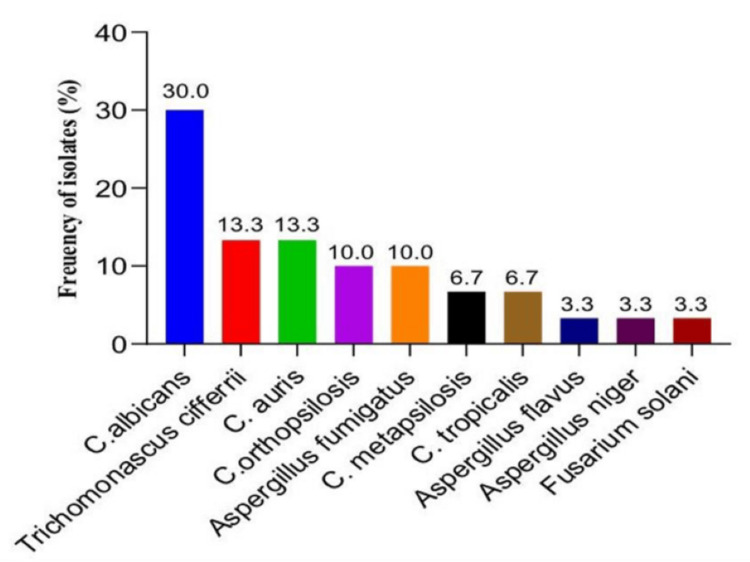
Species-wise distribution of culture-positive samples (n = 30). This image was created by Dr. Farha Siddiqui using Microsoft PowerPoint (Microsoft Corporation, Redmond, WA, USA).

The study evaluated 136 clinical samples in total. Of these, 12 (44.4%) did not produce fungal growth on culture, whereas 27 (19.9%) tested positive on direct microscopy using KOH mount. Among the culture-negative samples, 15 (50.0%) had negative KOH microscopy results, while 30 (22.1%) samples had positive fungal cultures. Overall, 30.9% (42/136) of the patients had evidence of fungal involvement (prevalence) based on microscopy and culture; the samples, comprising 63 (46.3%) and 73 (53.7%), respectively, were taken from the inpatient department (IPD) and outpatient department (OPD). Of the 30 culture-positive cases, 6 (20%) and 24 (80%) were moulds and yeast, respectively. NAC isolates (n = 15) were more prevalent than *Candida albicans* (n = 9), as Figure 2 illustrates.

**Figure 3:**
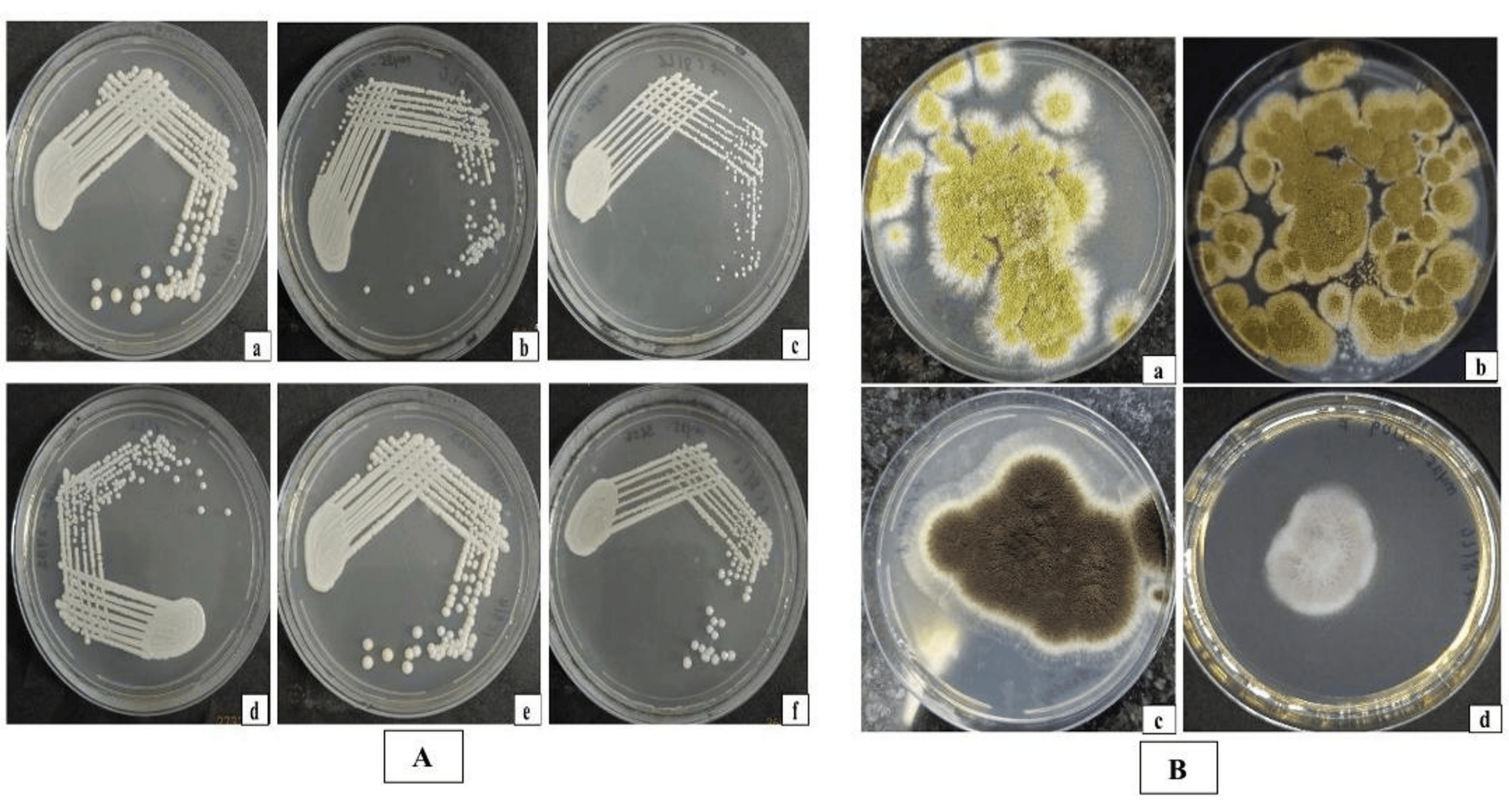
Growth of the fungal isolate species. (A) Yeasts including: (a) *Candida albicans*, (b) *Candida auris*, (c) *Candida parapsilosis*, (d) *Candida metapsilosis*, (e) *Candida tropicalis*, and (f) *Trichomonascus ciferrii*. (B) Moulds including: (a) *Aspergillus flavus*, (b) *Aspergillus fumigatus*, (c) *Aspergillus niger*, and (d) *Fusarium* spp.

Among the 30 culture-positive isolates, *C. albicans* was the most prevalent yeast species (9, 30%). *Trichomonascus ciferrii* and *C. auris* each accounted for four isolates (13.3%). Other yeasts included *Candida tropicalis* (2, 6.7%), *Candida orthopsilosis* (3, 10.0%), and *Candida metapsilosis* (2, 6.7%). Among mould isolates, *Aspergillus fumigatus* (3, 10.0%) was the most prevalent species, followed by *Aspergillus flavus* (1, 3.3%), *Aspergillus niger* (1, 3.3%), and *Fusarium solani* (1, 3.3%) (Figure 2). The data represent a wide range of fungal infections, with *Candida* spp. accounting for the majority of culture-positive cases. The growth of the fungal isolates is shown in Figures 3A-3B.

Table 1 summarizes the study participants' clinical and demographic attributes. Of the 136 patients, 30 (22.1%) had a fungal infection, while 106 (77.9%) did not. The age distribution and gender of the two groups did not differ statistically significantly (p = 0.3359 and p = 0.3812, respectively). Among clinical features, hearing loss was the only symptom significantly associated with fungal infection, observed in 30% of patients in the fungal group compared to 9.4% in the non-fungal group (p = 0.006). Other symptoms, such as ear pain (33.3% vs. 31.1%; p = 0.827), vertigo (26.7% vs. 18.9%; p = 0.359), and headache (23.3% vs. 26.4%; p = 0.742), were slightly more or variably distributed between groups but did not show statistical significance. A history of prior antibiotic intake was noted in 20% of fungal cases and 17.9% of non-fungal cases (p = 0.793), suggesting no significant association. Likewise, a higher proportion of patients with fungal infection reported similar complaints among family members (40% vs. 27.4%), although this difference did not reach statistical significance (p = 0.178). Overall, these results indicate that fungal involvement in CSOM was substantially correlated with clinical symptomatology, even though demographic characteristics were similar across groups. With respect to risk factors, none showed a statistically significant association with fungal infection. A history of steroid intake was present in 16.7% of fungal cases and 10.4% of non-fungal cases (p = 0.342). Diabetes mellitus was observed in 13.3% and 18.9% of fungal and non-fungal groups, respectively (p = 0.481). Malnutrition was reported in 13.3% of fungal cases compared to 17.0% of non-fungal cases (p = 0.641), while malignancy was rare in both groups (3.3% vs. 3.8%; p = 1.000).

**Table 1:** Clinical and demographic characteristics of the recruited study participants (n = 136). Note: Values are expressed as a number (n) and a percentage (%). Statistical significance is denoted as p < 0.05, where * indicates a significant difference between the CSOM patients with fungal infections and the CSOM patients without fungal infections. Fisher’s Exact Test was used for variables with small cell counts. Composite p-value for overall symptom comparison: 0.0022. † symbol denotes statistically significant value (<0.05). CSOM: Chronic suppurative otitis media

Clinical-Demographic Details	Parameters	CSOM With Fungal Infection (n = 30), n (%)	CSOM Without Fungal Infection (n = 106), n (%)	p-value
Age distribution	<18 yrs	6 (20%)	19 (17.9%)	0.336
	≥18 yrs	24 (80%)	87 (82.1%)	
Gender distribution	Female	12 (40%)	39 (36.8%)	0.381
	Male	18 (60%)	67 (63.2%)	
Clinical signs and symptoms*	Ear pain	10 (33.3%)	33 (31.1%)	0.827
	Hearing loss	9 (30%)	10 (9.4%)	0.006†
	Vertigo	8 (26.7%)	20 (18.9%)	0.359
	Headache	7 (23.3%)	28 (26.4%)	0.742
	History of antibiotic intake before testing	6 (20%)	19 (17.9%)	0.793
	Similar complaint in the family	12 (40%)	29 (27.4%)	0.178
Risk factors*	History of steroid intake	5 (16.7%)	11 (10.4%)	0.342
	Diabetes mellitus	4 (13.3%)	20 (18.9%)	0.481
	Malnutrition	4 (13.3%)	18 (17.0%)	0.641
	Malignancy	1 (3.3%)	4 (3.8%)	1.000

The minimum inhibitory concentrations (MICs) of various antifungal drugs were assessed for mould and yeast isolates collected throughout the investigation. The isolates were classified as either WT or NWT, based on the available ECVs for antifungal drugs for which clinical breakpoints (CBPs) were not available for any species. Results were presented as GM MICs when neither CBP nor ECVs were established. Fluconazole (0.12-256 µg/mL), voriconazole (0.008-8 µg/mL), posaconazole (0.008-8 µg/mL), itraconazole (0.008-8 µg/mL), amphotericin B (0.12-8 µg/mL), caspofungin (0.008-8 µg/mL), anidulafungin (0.015-8 µg/mL), micafungin (0.008-8 µg/mL), and 5-flucytosine (0.06-64 µg/mL) were the antifungal agents tested over the indicated concentration ranges (Table 2).

**Table 2:** Antifungal susceptibility profiles of Candida spp. isolates for which interpretative breakpoints are available (n = 24). Note: Values are expressed as a number (n) and a percentage (%). *Geometric mean was calculated for the antifungal-isolate combination, **antifungals for which ECV was available against a specific isolate. I, Intermediate; R, Resistant; S, Susceptible; SDD, Susceptible dose-dependent; GM, Geometric mean; ECV, Epidemiological cutoff; WT, Wild type; NWT, Non-wild type

Antifungal Medication	*Candida albicans,* n = 9, N(%)	*Candida* *auris,* n = 4, N(%)	*Trichomonascus ciferrii,* n = 4, N(%)	*Candida* *orthopsilosis,* n = 3, N(%)	*Candida* *tropicalis, *n = 2, N(%)	*Candida* *metapsilosis, *n = 2, N(%)
Fluconazole						
S	5 (55.56)	0 (0.00)	-	2 (66.67)	1 (100.00)	2 (100.00)
SDD	0 (0.00)	0 (0.00)	-	0 (0.00)	0 (0.00)	0 (0.00)
I	0 (0.00)	0 (0.00)	-	1 (33.33)	0 (0.00)	0 (00.00)
R	4 (44.44)	4 (100.00)	-	0 (00.00)	1 (10.00)	0 (00.00)
*GM	-	-	1.17	-	-	-
Anidulafungin						
S	9 (100.00)	4 (100.00)	-	3 (100.00)	2 (100.00)	2 (100.00)
I	0 (0.00)	0 (0.00)	-	0 (0.00)	0 (0.00)	0 (0.00)
R	0 (0.00)	0 (0.00)	-	0 (00.00)	0 (00.00)	0 (0.00)
*GM	-	-	0.48	-	-	-
Caspofungin						
S	9 (100.00)	3 (75.00)	-	3 (100.00)	2 (100.00)	2 (100.00)
I	0 (0.00)	0 (0.00)	-	0 (0.00)	0 (0.00)	0 (0.00)
R	0 (0.00)	1 (25.00)	-	0 (00.00)	0 (00.00)	0 (00.00)
*GM	-	-	0.05	-	-	-
Micafungin						
S	9 (100.00)	4 (100.00)	-	3 (100.00)	2 (100.00)	2 (100.00)
I	0 (0.00)	0 (0.00)	-	0 (0.00)	0 (0.00)	0 (0.00)
R	0 (0.00)	0 (0.00)	-	0 (00.00)	0 (00.00)	0 (0.00)
*GM	-	-	0.20	-	-	-
Voriconazole						
S	4 (44.45)	0 (0.00)	-	3 (100.00)	0 (00.00)	2 (100.00)
I	3 (33.33)	0 (0.00)	-	0 (0.00)	1 (50.00)	0 (0.00)
R	2 (22.22)	4 (100.00)	-	0 (00.00)	1 (50.00)	0 (0.00)
*GM	-	-	0.34	-	-	-
Posaconazole						
*GM	2.04	0.6	0.34	0.21	0.6	0.06
* Itraconazole						
*GM	-	0.3	0.24	-	-	-
WT	4	-	-	3	1	2
NWT	5	-	-	0	1	0
** 5-Flucytosine						
*GM	0.08	32	38.05	-	-	-
WT	-	-	-	3	1	1
NWT	-	-	-	0	1	0
** Amphotericin B						
S	-	4 (100.00)	-	-	-	-
I	-	0 (0.00)	-	-	-	-
R	-	0 (0.00)	-	-	-	-
*GM	-	-	1	-	-	-
WT	9	-	-	3	1	2
NWT	0	-	-	0	1	0

Overall yeast susceptibility patterns

Echinocandins showed remarkable in vitro activity against all *Candida* species, with 100% susceptibility to anidulafungin and micafungin. Except for one isolate of *C. auris*, all species remained 100% susceptible to caspofungin.

Four (44.4%) of the *C. albicans* isolates (n = 9) were resistant to fluconazole, whereas five (55.6%) were sensitive. All *C. auris* isolates (n = 4) were 100% resistant to fluconazole. Fluconazole susceptibility was maintained in *C. orthopsilosis* (3/2, 66.7%), *C. tropicalis* (1/2, 50.0%), and *C. metapsilosis* (2/2, 100%) among NAC species. Overall, NAC species - especially *C. auris* - were more likely to exhibit fluconazole resistance, indicating that fluconazole's in vitro efficacy against newly developing *Candida* infections was diminished.

Based on the available CLSI breakpoints, all four isolates of *C. auris* were classified as amphotericin B-sensitive. According to the current study, amphotericin B shows strong in vitro activity against all *Candida* species, including multidrug-resistant *C. auris*, suggesting it may remain helpful in treating fungal infections associated with CSOM.

Interpretation of *Candida species* using ECV

For itraconazole, among *C. albicans* isolates (n = 9), four (44.4%) were classified as WT (ECV ≤ 0.12 µg/mL), while five (55.6%) were categorized as NWT (ECV > 0.12 µg/mL). All *C. orthopsilosis* isolates (n = 3) were WT (ECV ≤ 0.25 µg/mL). Among *C. tropicalis* isolates (n = 2), one isolate was WT (ECV ≤ 0.5 µg/mL), and one was NWT (ECV > 0.5 µg/mL). Both *C. metapsilosis* isolates (n = 2) were WT (ECV ≤ 0.25 µg/mL). Similarly, in another set of *C. orthopsilosis* isolates (n = 3), all were WT (ECV ≤ 0.25 µg/mL).

For 5-flucytosine, all *C. orthopsilosis* isolates (n = 3) were WT (ECV ≤ 0.5 µg/mL). Among *C. tropicalis* isolates, one was WT (ECV ≤ 0.5 µg/mL), and one was NWT (ECV > 0.5 µg/mL), while both *C. metapsilosis* isolates were WT (ECV ≤ 0.5 µg/mL).

For amphotericin B, all *C. albicans* isolates (n = 9) and *C. orthopsilosis* isolates (n = 3) were WT (ECV ≤ 2 µg/mL). Among *C. tropicalis* isolates, one was WT (ECV ≤ 2 µg/mL), and one was NWT (ECV > 2 µg/mL). Both *C. metapsilosis* isolates were WT (ECV ≤ 2 µg/mL).

Geometric mean minimum inhibitory concentrations (GM MICs)

T. ciferrii

*T. ciferrii* (n = 4) AFST results were reported using GM MICs due to the lack of defined breakpoints and ECVs. Individual MICs are also included in Table 3: amphotericin B, 1.0; caspofungin, 0.05; anidulafungin, 0.48; micafungin, 0.2; and 5-flucytosine, 38.05. Fluconazole, 1.17; voriconazole, 0.34; posaconazole, 0.34; itraconazole, 0.24; and 5-flucytosine, 38.05.

**Table 3:** MIC value of individual Candida spp. (n = 24). FZ, Fluconazole; VOR, Voriconazole; PZ, Posaconazole; IZ, Itraconazole; AB, Amphotericin B; CAS, Caspofungin; AND, Anidulafungin; MF, Micafungin; FC, Flucytosine; MIC, Minimum inhibitory concentration

Isolates	FZ (MIC)	VOR (MIC)	PZ (MIC)	IZ (MIC)	AB (MIC)	CAS (MIC)	AND (MIC)	MF (MIC)	FC (MIC)
Candida albicans	0.5	0.015	0.03	0.06	0.03	0.06	0.03	0.008	0.06
	0.5	0.008	0.03	0.06	1	0.12	0.03	0.015	0.06
	0.25	0.008	0.015	0.03	1	0.06	0.015	0.008	0.06
	16	0.25	0.25	0.5	0.5	0.03	0.015	0.015	0.12
	32	0.5	2	0.5	1	0.03	0.015	0.03	0.06
	>128	>8	>8	>16	1	0.06	0.06	0.03	0.12
	>128	>8	>8	>16	1	0.06	0.06	0.03	0.12
	0.5	0.015	0.03	0.12	1	0.03	0.03	0.008	0.12
	0.5	0.25	0.03	0.5	1	0.03	0.015	0.12	0.06
Candida auris	>256	4	0.25	0.25	1	0.25	0.25	0.12	0.25
	>256	8	0.25	0.5	1	>8	0.12	0.25	64
	256	8	2	0.5	2	8	0.25	0.25	64
	>256	4	0.06	0.12	1	0.12	0.12	0.12	0.06
Trichomonascus cifferrii	64	0.5	0.5	0.25	1	0.06	>8	0.25	2
	32	1	2	1	1	0.12	0.06	0.015	0.06
	64	0.5	0.25	0.12	1	0.03	0.12	0.12	1
	16	0.06	0.06	0.12	1	0.03	1	0.06	16
Candida orthopsilosis	4	0.25	0.25	0.12	1	0.5	0.25	0.5	0.12
	2	0.25	0.25	0.12	1	0.5	0.25	0.5	0.06
	1	0.03	0.12	0.25	1	0.5	1	0.5	0.06
Candida tropicalis	32	1	1	1	4	0.06	0.015	0.12	8
	2	0.25	0.25	0.25	2	0.12	0.015	0.03	0.06
Candida metapsilosis	2	0.06	0.06	0.25	0.25	0.25	0.5	0.5	0.06
	4	0.06	0.06	0.12	1	0.25	0.12	0.25	0.06

Other Fungal Isolates

Among *C. albicans* isolates (n = 9), the GM MIC for posaconazole was 2.04 µg/mL. In contrast, lower GM MICs were observed among NAC species, including *C. auris* (0.6 µg/mL), *C. orthopsilosis* (0.21 µg/mL), *C. tropicalis* (0.6 µg/mL), and *C. metapsilosis* (0.06 µg/mL). For itraconazole, the GM MIC for *C. auris* was 0.3 µg/mL. For 5-flucytosine, the GM MIC for *C. albicans* was 0.08 µg/mL, and for *C. auris*, it was 32.08 µg/mL.

AFST of mould isolates

**Figure 4:**
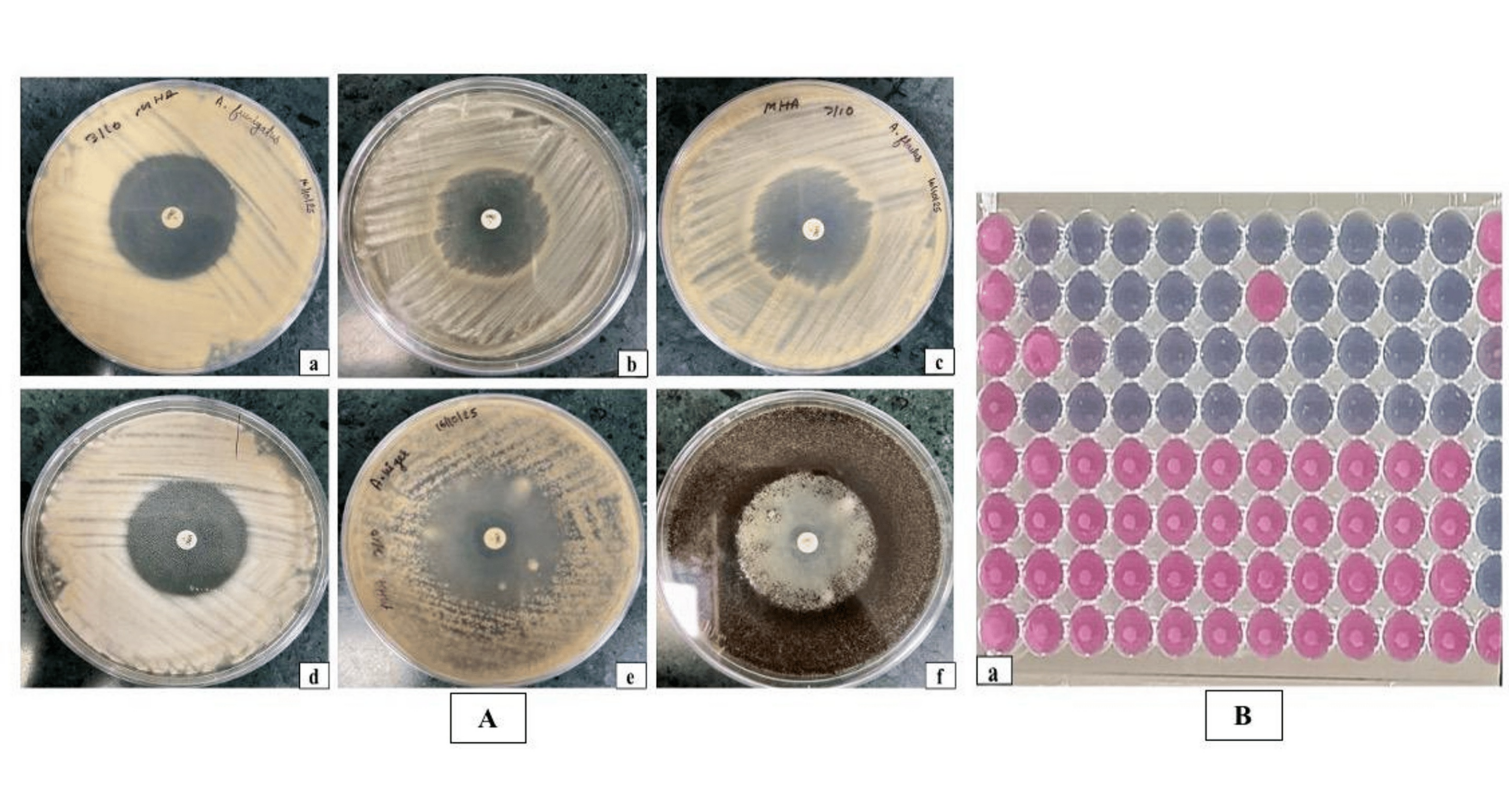
(A) In vitro susceptibility of mould isolates to voriconazole (disk diffusion, 5 µg disk): (a) Aspergillus flavus - 39 mm; (b-d) Aspergillus fumigatus - 33, 35, and 31 mm; (e) Fusarium solani - 19 mm; (f) Aspergillus niger - 32 mm. (B) Antifungal susceptibility testing (AFST) for yeast isolates, performed using the Sensititre™ YeastOne® colorimetric broth microdilution method.

Voriconazole (5 µg) disk diffusion was used to assess the AFST of mould isolates. Of all the *Aspergillus* species, *A. flavus* displayed the largest zone of inhibition (39 mm). *A. fumigatus* isolates revealed inhibitory zones ranging from 31 to 35 mm, whilst *A. niger* showed a zone diameter of 32 mm. This suggests that all *Aspergillus* species are highly susceptible to voriconazole. *F. solani*, on the other hand, showed a significantly narrower zone of inhibition (19 mm), suggesting decreased susceptibility to voriconazole (Figure 4A). The significance of species-specific AFST testing in mould-related diseases is highlighted by the fact that *F. solani* had a restricted response to voriconazole. In contrast, *Aspergillus* species generally displayed favourable in vitro sensitivity.

## Discussion

In low- and middle-income nations, environmental factors, delayed access to healthcare, and recurrent exposure to antibiotics all contribute to the chronicity and treatment failure of CSOM, which continues to be a significant public health concern. There is growing evidence that fungal pathogens contribute to persistent CSOM in a clinically meaningful way. Fungal involvement was observed in 30.9% of clinically diagnosed CSOM cases in the current investigation, underscoring the importance of mycological assessment in chronic ear infections.

The global prevalence of CSOM was estimated by Onifade et al. in a recent systematic study to be 3.8% [2]; however, this figure represents population-level prevalence rather than fungal positivity among verified CSOM patients. *Aspergillus* spp. are typically responsible for fungal isolation rates ranging from roughly 13% to over 30%, according to Indian research [14,15]. Our results fall towards the higher end of this range. These findings highlight the clinical significance of fungal infection, especially in humid, resource-constrained environments, in chronic, treatment-refractory CSOM.

The discrepancy between fungal culture and direct KOH microscopy was a noteworthy finding. While several culture-positive samples were KOH-negative, several KOH-positive samples were unable to grow fungus. This result confirms previous studies that highlighted the complementary functions of culture and microscopy in the diagnosis of fungal CSOM [16]. These differences could be explained by variables such as low fungal burden, sampling variability, nonviable organisms, and previous exposure to antifungals. The combined use of microscopy and culture to increase diagnostic yield is therefore recommended, as relying solely on one diagnostic modality may understate fungal involvement.

Moulds were less common than yeasts, which accounted for 80% of culture-positive cases. This result is consistent with recent research from tropical and Indian regions that shows a growing contribution of *Candida* spp. to chronic ear infections [15]. *C. albicans* remained the most frequent isolate, while NAC species prevailed overall, indicating a changing epidemiological trend, as previously shown [17]. *T. ciferrii* (formerly *Stephanoascus ciferrii*), although rare, has been increasingly reported in chronic ear infections and is often associated with antifungal resistance. The isolation of *C. auris* and *T. ciferrii* is noteworthy because it highlights the emergence of uncommon and potentially resistant yeasts in CSOM. Their detection highlights how fungal aetiology is changing and how clinical management must be guided by accurate species identification and tailored antifungal therapy [18]. In line with previous research, *A. fumigatus* remained the most commonly isolated mould species.

Fungal CSOM was strongly associated with more severe symptoms, especially hearing loss, consistent with other findings linking fungal infection to mucosal damage and chronic inflammation [15]. Conversely, as other researchers have found, fungal infection was not substantially correlated with demographic characteristics or assessed risk factors such as diabetes mellitus and steroid use [19]. These findings support routine mycological examination in chronic or symptomatic cases and imply that fungal CSOM is not limited to conventionally identified high-risk populations.

In line with surveillance data from India and around the world, AFST testing showed high rates of fluconazole resistance, especially among *C. auris* and NAC spp. [20,21]. NAC spp.' varying azole susceptibility patterns were similar to those found in previous Indian investigations [22]. On the other hand, amphotericin B maintained good efficacy, including against *C. auris*. At the same time, echinocandins demonstrated strong in vitro activity against all *Candida* isolates, suggesting their continued use in refractory or azole-resistant infections [23-25]. Significant therapeutic challenges are posed by *C. auris*, a rapidly emerging multidrug-resistant yeast increasingly identified in CSOM. Accurate identification and antifungal-susceptibility-guided care are crucial to preventing therapy failure. *Aspergillus* spp. showed good susceptibility to voriconazole among moulds [26], according to Indian and international research, but *F. solani* demonstrated lower susceptibility, consistent with its well-known inherent resistance to azoles [27,28].

Overall, these findings highlight the significance of accurate species identification and susceptibility-guided treatment to optimize therapeutic outcomes and demonstrate how the mycological spectrum and antifungal resistance patterns in CSOM are evolving. This study's strengths include thorough AFST for yeasts and moulds, systematic sampling, and accurate species identification using MALDI-TOF MS. Limitations include the use of disk diffusion for mould susceptibility testing, the single-centre design, minimal species-wise sample sizes, and the absence of defined CBPs/ECVs for rare species that require GM MIC reporting.

## Conclusions

In over one-third of cases, fungal infections are a key factor in CSOM. Echinocandins and amphotericin B continue to work, but yeasts - particularly NAC species like *C. auris* and *T. ciferrii - *are the most common and often exhibit significant azole resistance. Unlike *Aspergillus* species, which are frequently susceptible to voriconazole, *F. solani* is less vulnerable. The many distinctions between KOH microscopy and culture underscore the importance of combining diagnostic approaches. Fungal CSOM is associated with more severe symptoms, particularly hearing loss, emphasizing the need for routine mycological evaluation, accurate species identification, and AFST-guided therapy for optimal management, especially in resource-limited settings.
